# Combined Resistance to Ceftolozane-Tazobactam and Ceftazidime-Avibactam in Extensively Drug-Resistant (XDR) and Multidrug-Resistant (MDR) *Pseudomonas aeruginosa*: Resistance Predictors and Impact on Clinical Outcomes Besides Implications for Antimicrobial Stewardship Programs

**DOI:** 10.3390/antibiotics10101224

**Published:** 2021-10-08

**Authors:** Marianna Meschiari, Gabriella Orlando, Shaniko Kaleci, Vincenzo Bianco, Mario Sarti, Claudia Venturelli, Cristina Mussini

**Affiliations:** 1 Infectious Disease Clinic, Policlinico University Hospital, 41122 Modena, Italy; meschiari.marianna@aou.mo.it (M.M.); cristina.mussini@unimore.it (C.M.); 2 Clinical and Experimental Medicine, University of Modena and Reggio Emilia, 41122 Modena, Italy; shaniko.kaleci@unimore.it; 3 Infectious Disease Clinic, Cotugno Hospital, 80131 Naples, Italy; bianco.vincenzo@yahoo.it; 4 Clinical Microbiology Laboratory, University of Modena and Reggio Emilia, 41122 Modena, Italy; sarti.mario@aou.mo.it (M.S.); venturelli.claudia@aou.mo.it (C.V.)

**Keywords:** MDR/XDR *Pseudomonas aeruginosa*, ceftazidime/avibactam, ceftolozane/tazobactam, combined resistance to ceftazidime/avibactam and ceftolozane/tazobactam, difficult to treat pathogen, hospital acquired infections, carbapenems use, combining antimicrobial stewardship and infection control

## Abstract

A retrospective case-control study was conducted at Modena University Hospital from December 2017 to January 2019 to identify risk factors and predictors of MDR/XDR *Pseudomonas aeruginosa* (PA) isolation with resistance to ceftazidime/avibactam (CZA) and ceftolozane/tazobactam (C/T), and of mortality among patients infected/colonized. Among 111 PA isolates from clinical/surveillance samples, 60 (54.1%) were susceptible to both drugs (S-CZA-C/T), while 27 (24.3%) were resistant to both (R-CZA-C/T). Compared to patients colonized/infected with S-CZA-C/T, those with R-C/T + CZA PA had a statistically significantly higher Charlson comorbidity score, greater rate of previous PA colonization, longer time before PA isolation, more frequent presence of CVC, higher exposure to C/T and cephalosporins, longer hospital stay, and higher overall and attributable mortality. In the multivariable analysis, age, prior PA colonization, longer time from admission to PA isolation, diagnosis of urinary tract infection, and exposure to carbapenems were associated with the isolation of a R-C/T + CZA PA strain, while PA-related BSI, a comorbidity score > 7, and ICU stay were significantly associated with attributable mortality. C/T and CZA are important therapeutic resources for hard-to-treat PA-related infections, thus specific antimicrobial stewardship interventions should be prompted in order to avoid the development of this combined resistance, which would jeopardize the chance to treat these infections.

## 1. Introduction

On February 2017, the WHO published a list of antibiotic-resistant “priority pathogens” to push the research and development of new and effective antibiotic treatments [1]. Carbapenem-resistant *Pseudomonas aeruginosa* (PA) was therefore ranked as one of the most critical pathogens because of its propensity to develop an MDR/XDR/PDR phenotype according to the CDC definition [2] by accumulating resistance to nearly all-available antibiotics by several different mechanisms, mainly concerning the selection of mutations in chromosomal genes and the acquisition of transferable resistance determinants [3,4]. The appearance of MDR/XDR/PDR PA requires identifying strategies that would prevent hospitalized patients from acquiring the bacterium by implementing infection control and prevention policies (ICP) [5,6]. Furthermore, PA remains as one of the major causes of healthcare-associated infections (HAIs) in Europe [7,8], leading to high morbidity and mortality rates due to limited therapeutic options [9]. As a response to the WHO call to provide new drugs for difficult-to treat PA, two novel β-lactam–β-lactamase inhibitor combinations (BLBLICs), namely ceftazidime/avibactam (CZA) and ceftolozane/tazobactam (C/T), have been commercialized. Avibactam is a non-b-lactam b-lactamase inhibitor active against Ambler class A KPC, ESBLs and class C AmpCs, while ceftolozane is a ceftazidime-derived novel cephalosporin, which is not hydrolyzed by ESBLs and AmpCs [10]. As could be expected, soon after their launch, several reports of resistance to these new BLBLICs were reported, highlighting the need to provide comprehensive data on the risk factors for the development of resistance [11,12,13]. In this very complex scenario, to balance the access to antibiotics, optimizing the use of the new BLBLICs with the control of antibiotic resistance is a global public health priority [14,15]. The aim of our study was to describe patients who acquire an infection/colonization due to an MDR/XDR-PA with combined resistance to C/T and CZA; to characterize those isolates; and to further investigate the impact of this resistance on patients’ clinical outcomes. 

## 2. Results

Between December 2017 and January 2019, 111 consecutive patients with an MDR-PA isolated from a clinical or surveillance sample were included in the study. Among the isolates, 38.8% (43/111) and 1.8% (2/111) exhibited an XDR and PDR phenotype, respectively. Sixty (54.1%) PA isolates were defined as S-CZA-C/T (susceptible to both CZA and C/T), 27 (24.3%) as R-CZA-C/T (resistant to both CZA and C/T), 12 (10.8%) as R-C/T (resistant to C/T only), and 12 (10.8%) as R-CAZ (resistant to CZA only). Prevalence of XDR and PDR phenotypes in the S-CZA-C/T and R-CZA-C/T groups were 51.6% (31 isolates) and 3.3% (two isolates), and 62.9% (16 strains) and 3.7% (one strain), respectively.

### 2.1. Antibiotic Susceptibility

**Figure 1:**
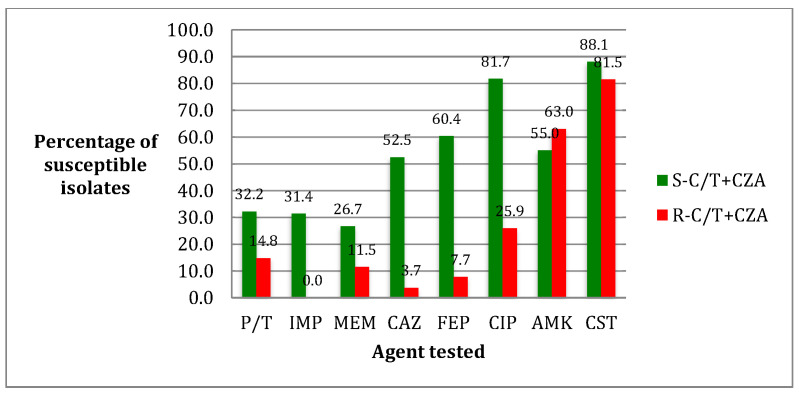
Comparison of susceptibility rates between S-C/T + CZA and R-C/T + CZA PA isolates. Abbreviations: P/T: piperacillin/tazobactam; IMP: imipenem; MEM: meropenem; CAZ: ceftazidime; FEP: cefepime; CIP: ciprofloxacin; AMK: amikacin; and CST: colistin.

Regarding single antibiotic susceptibility against all PA isolates, colistin was active in 90.9% (101/111) of cases, amikacin in 82.8% (92/111), ciprofloxacin in 38.7% (43/111), while CZA and C/T susceptibility rates were 67.6% (75/111) and 66.6% (74/111), respectively. Among anti-pseudomonal beta-lactams, cefepime exhibited the best susceptibility level at 38.2% (39/102), followed by ceftazidime at 30.6% (34/111), imipenem at 26.3% (15/57), and piperacillin/tazobactam (P/T) at 24.3% (27/111). The lowest susceptibility rate of the entire panel was that of meropenem with 20.7% (23/111). Considering PA strains resistant to beta-lactams (beta-lactam-R 38/111) and to both beta-lactams and ciprofloxacin (beta-lactam-R/CIP-R 28/111), C/T and CZA susceptibility rates in the two groups were 42.1% (16/38) and 42.9% (12/28), and 39.5% (15/38) and 42.9% (12/28), respectively. Interestingly, 28.9% of R-CZA strains were susceptible to C/T, while 30.7% of R-C/T isolates maintained susceptibility to CZA. Figure 1 illustrates the comparison of susceptibility rates between S-C/T + CZA and R-C/T + CZA PA isolates; a statistically significant difference was found for imipenem (31.4% vs. 0%; *p* = 0.033), ceftazidime (52.5% vs. 3.7%; *p* < 0.0001), and cefepime (60.4% vs. 7.7%; *p* < 0.0001).

### 2.2. Characteristics of Patients Colonized/Infected with a S-C/T + CZA PA and R-C/T + CZA PA

Characteristics of patients colonized/infected with a S-C/T + CZA PA and R-C/T + CZA PA are described in Table 1. The two populations differed significantly for some characteristics and particularly those with a R-C/T + CZA PA had higher Charlson Comorbidity Index (CCI) scores, were more frequently previously colonized by PA, had longer hospitalization and ICU stays, and more frequently had a central venous catheter (CVC). Considering previous antibiotic exposure, they had been treated more frequently with C/T and with third to forth generation cephalosporins (3-4GC). Concerning outcomes, patients with R-C/T + CZA PA showed a longer length of hospital stay (LOS) as well as both a higher crude and attributable mortality, although not statistically significant. Seventy-six patients (65%) had been diagnosed with a PA-related infection (pneumonia, 34% of cases; ABSSSI, 16%; IAI, 15%; UTI, 11%; BSI, 9%; PJI, 5%; and CNS, 4%), while in the remaining 35 patients, PA was defined as a colonizer (twenty among them were infected with another pathogen). Twenty patients (74.1%) in the R-C/T + CZA group and 34 (57.6%) in the S-C/T + CZA group had a PA-related infection. There was no statistically significant difference in the percentage of PA-colonization or PA-related different types of infection between patients in the S-C/T + CZA group and those in the R-C/T + CZA group.

### 2.3. Risk Factors for Combined Resistance to C/T and CZA 

Table 2 shows that age, prior colonization with PA, longer time from admission to PA isolation, the presence of a urinary tract infection (UTI), and previous exposure to carbapenems (CA) were risk factors significantly associated with the isolation of a R-C/T + CZA PA strain in the multivariable analysis. 

### 2.4. Survival Rate Analysis and Predictors of Mortality

**Figure 2:**
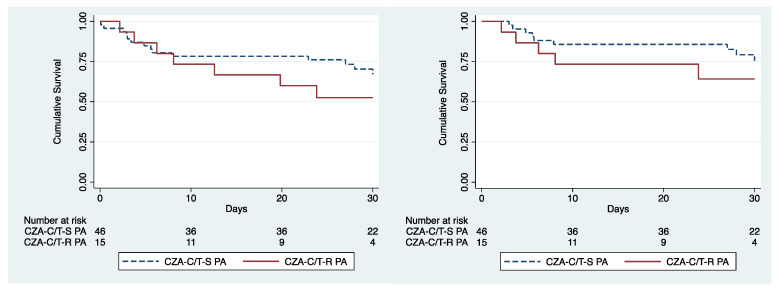
Survival curve showing overall (left panel) and attributable 30-day mortality (right panel) in patients colonized/infected with S-CZA-C/T vs. R-CZA-C/T-R PA.

The 30-day overall mortality rate among R-CZA-C/T PA patients was 53% compared to 44% in patients with S-CZA-C/T PA (*p* = 0.358). Infectious cause-attributable 30-day mortality was 43% and 36% in R-CZA-C/T PA and S-CZA-C/T PA, respectively (*p* = 0.383) (Figure 2). The univariate analysis showed that older age, the diagnosis of a PA-related BSI, CCI score mean and CCI ≥ 7, the days of time at risk, LOS in ICU, the presence of a CVC, the presence of a urinary catheter (UC), and the combined resistance to C/T and CZA were significantly associated with 30-day attributable mortality (data not shown). In the multivariate analysis, risk factors significantly associated to 30-day attributable mortality were PA-related BSI, CCI > 7, and LOS in ICU (Table 3).

## 3. Discussion

Our study describes a cohort of 111 patients infected/colonized with an MDR/XDR-PA strain, in which almost twenty-five percent of cases were resistant to both new BLBLICs just one year after their introduction for routine hospitals use. To our knowledge, this is one of the few studies attempting to identify risk factors for combined resistance to both CZA and C/T in MDR/XDR-PA strains, as well as predictors of mortality in this peculiar sub-set of patients.

Many studies have focused on singular CZA and C/T resistance rates in PA in vitro, showing that PA resistance rates to C/T and CZA are higher when considering MDR and XDR strains, for which these new drugs are usually considered as first line therapies [16,17,18,19,20,21,22] 

In our study, those PA strains resistant to beta-lactams had C/T and CZA susceptibility rates of 42.1% and 42.9%, respectively, while we also explored the residual susceptibility between the new two BL/BLICs. Among C/T-resistant isolates, CZA retained a 30.7% susceptibility rate, while among CZA-resistant isolates, C/T preserved a 28.9% susceptibility level. These are important data because, in the case of isolate resistance to one of the two drugs, clinicians have almost a 30% chance of using the active left one. 

Indeed, our data are quite in line with those recently published by Sid Ahmed MA, et al. who prospectively evaluated 205 MDR-PA strains. Sixty-eight percent were susceptible to CZA, 62.9% were susceptible to C/T, 59.0% were defined as S-C/T + CZA, and 27.3% were defined as R-C/T + CZA. Twenty (9.8%) isolates were susceptible to CZA but not to C/T and only eight (3.9%) were susceptible to C/T but not to CZA. Less than 50% of XDR isolates were susceptible to CZA or C/T [23]. 

In our cohort, prior exposure to CZA and/or C/T was not an independent risk factor for the R-C/T + CZA PA phenotype but this result could be explained by the very limited use of the new BLBLICs at our center at the time of data collection. 

Tamma PD et al. analyzed the emergence of resistance to C/T after C/T therapy in 28 consecutive patients infected with CA-non-susceptible PA. Fifty percent of strains became C/T non-susceptible and the main risk factors were inadequate source control and use of C/T not in the 3-hour infusion. Quite interesting 86% of these isolates, which were in the beginning susceptible to CZA, acquired high-level resistance to CZA (without any exposure) after C/T use [24]. This datum has been recently confirmed by another paper showing that treatment-emergent resistance to C/T was very frequently associated with CZA cross-resistance [25].

The main strength of our study is that it includes correlated patients’ clinical characteristics, including antibiotic exposure, to the emergence of CZA and C/T combined resistance. Accordingly, age, prior colonization with PA, longer time from admission to PA isolation, the diagnosis of a PA-related UTI, and previous exposures to CA significantly predicted the presence of a R-C/T + CZA PA strain. All these variables describe the clinical picture of a complex patient in which the choice of the best antibiotic to use poses several therapeutic challenges. Considering our data, forty percent of patients with a R-C/T + CZA PA had been exposed to CA in the previous 6 months and prior CA exposure almost doubled the risk of developing combined resistance to the new BLBLICs, even without their direct exposure. We believe that this finding deserves careful consideration. The role of CA exposure as a risk factor for infections due to MDR-PA has been already investigated [26,27,28] but, to our knowledge, this is the first study which suggests that CA exposure is associated with the evolution of PA resistance to the new BLBLICs. 

Considering the still on-going discussion about the best therapy for infections due to ESBL-producing bacteria between meropenem and BLBLICs [29], our data seem to suggest that, in particular, in clinical settings in which MDR-PA or OXA-48- and KPC-producer bacteria are highly prevalent, the use of meropenem should be carefully evaluated and tailored to the patient in order to reduce the risk of developing resistance to CZA and C/T that remain as important options for infections due to the above-mentioned resistant pathogens. 

In the survival analysis, the trend for lower survival in the R-C/T + CZA PA group did not reach statistical significance and combined resistance was not predictive of attributable mortality. In fact, combined resistance was a risk factor for mortality in the univariate analysis only, thus we hypothesize that the low number of patients in the R-C/T + CZA PA group did not allow us to reach statistical significance. 

However, we showed that mortality was predicted by the diagnosis of a PA-related BSI, a higher comorbidity score, and a longer LOS in ICU. These findings are concordant with several other papers [30,31]. 

Our study presents several limitations: it is a single center study; the total number of PA isolates was low when compared to previous large surveillance programs; and the retrospective nature of the study could underestimate the role of certain factors, whereas other factors not studied might have influenced the results. Moreover, risk factor analyses for antimicrobial resistance at the patient-level and population-level are prone to selection bias and ecological bias, respectively. Moreover, we did not perform an analysis of the mechanisms of resistance to CZA and C/T because it was beyond the aims of our study.

## 4. Materials and Methods

### 4.1. Study Design and Setting

This is a retrospective case-control study carried out at the Modena University Hospital, which is a 1200-lincesed bed public tertiary care hospital in Northern Italy, from December 2017 to January 2019. Since 2012, according to our infection control policy, all adults admitted to the hospital underwent a rectal swab to rule out carriage of carbapenem-resistant Gram-negative bacilli, including carbapenem-resistant PA (CRPA). In order to give an idea of the CRPA burden in our hospital, during the study period, between 12 and 16% of the total bloodstream infections were caused by CRPA and the incidence rate of MDR-PA (defined according to the CDC) [2] on December 2018 was 0.31 cases per 1000 bed days. The Institutional Ethics Committee of the University Hospital Policlinico Modena approved the study, but given the retrospective nature of the study, it was not possible to obtain written informed consent from the subjects enrolled due to organizational reasons. 

### 4.2. Definitions and Data Collection

PA strains with residual susceptibility to both new BLBLICs were defined as S-C/T + CZA; those resistant to both new BLBLICs were defined as R-C/T + CZA; those resistant to CZA only were defined as R-CZA; and those resistant to C/T only were defined as R-C/T. We included all consecutive non-duplicate patients admitted to the hospital with an MDR-PA isolate. XDR and PDR-PA isolates were defined using the CDC definition [2]. We applied NHSH/CDC criteria to define different PA-related infection types [31]. Colonization was defined as a positive PA isolation obtained from surveillance or screening cultures (stool, skin, mucous membranes, open wounds, excretions, or secretions) in the same patient within the previous six months, in the absence of relevant signs or symptoms suggestive of infection. Only one single isolate of MDR-PA per patient per analysis period was collected (both clinical samples and surveillance cultures). Isolates were selected with respect to their antimicrobial susceptibility profile and we chose the most resistant per patient as well as gave priority to diagnostic samples from sterile body sites. For patients with both an invasive and non-invasive positive culture, we considered only the invasive culture (including surveillance cultures in the absence of clinical ones). Additional information included: patients’ demographics (age and sex); underlying systemic disease (Charlson Comorbidity Index Score (CCI), divided in three severity classes: class 1 = score 0–3 points; class 2 = score 4–6 points; and class 3 = score ≥ 7 points); factors related to hospitalization (ICU stay, length of ICU stay, and time at risk, that is, days between admission and PA isolation); use of invasive devices (i.e., oro-tracheal intubation/tracheostomy tube (OTI/TT), central venous catheter (CVC), urinary catheter (UC); and previous PA colonization. We collected the residual sensitivity to all antibiotic and antibiotic exposure, the latter as a composite of any antibiotic use (“any antibiotic”) and as the exposure to a specific antibiotic class. We consulted the registry of drugs delivery provided by the hospital pharmacy and defined prior exposure as treatment with antimicrobials for at least 72 h during the 90-day period (CA, C/T, and CZA) and 30 days (FQ, 3-4GC, and P/T) prior to the MDR-PA isolation. Outcomes included: LOS, crude overall mortality (defined as death documented to have occurred within 90 days after colonization or infection with PA) and crude attributable mortality (death associated with a complicated PA infection during the same hospital stay), and 30-day overall and attributable mortality. All other clinical data were retrieved by direct consultation of the medical records.

### 4.3. Microbiological Methods

All non-duplicate PA isolates were selected with respect to their antimicrobial resistance profile based on microdilution antimicrobial susceptibility tests, choosing the most resistant isolates. All isolates were identified by MALDI-TOF MS using the VITEK MS (bioMérieux, Marcy l’Etoile, France) following the manufacturer’s instructions. The resistance profile was expressed by the different values of the minimum inhibitory concentration (MIC) determined by broth microdilution using the Micronaut S MDR MRGN-Screening 3 panel (MERLIN Diagnostika GmbH). PA isolates were considered non-susceptible to P/T, cefepime, ceftazidime, meropenem, amikacin, gentamicin, ciprofloxacin, colistin, C/T, and CZA if their MIC value was greater than the current EUCAST 2018 clinical breakpoint (susceptibility of PA to C/T and CZA for MIC values of ≤4 and ≤8 μg/dL, respectively). 

### 4.4. Statistical Analysis

Statistical analysis was performed using STATA® software version 14 (StataCorp. 2015. Stata Statistical Software: Release 14. College Station, TX: StataCorp LP). Descriptive statistics were presented for baseline demographic clinical characteristics for the entire group, as well as for the groups of patients. Continuous variables were presented as mean and standard deviation (SD), and the comparison between the subgroups was conducted using unpaired Student’s *t*-test for two groups; if the data did not follow the normal distribution, the Mann–Whitney test was used as an alternative and the results were presented as the median and 25th–75th percentile, while categorical variables were presented as frequency (N, %) and compared using Pearson’s chi-squared test. The association between these parameters and S-CZA-C/T PA vs. R-CZA-C/T PA or others was assessed using a logistic regression model with stepwise forward selection. A multivariate logistic regression model was carried out using a stepwise selection method to identify the prognostic factors between the groups. In the first step, the intercept-only model was fitted and individual score statistics for the potential variables were evaluated. A significance level of *p* < 0.05 was used to allow a variable into the model. In stepwise selection, an attempt was made to remove any insignificant variables from the model before adding a significant variable to the model. The Hosmer and Lemeshow tests were used to evaluate the “goodness of fit” in the selection model. Data from the univariate and multivariate logistic regression analyses were expressed as the odds ratio (OR) and 95% confidence interval (CI). The overall survival analysis (OS) and specific (attributable at the resistance to C/T and CAZ) survival (SS) at 30 days was estimated with the Kaplan–Meier method as well as compared using the log-rank test. The association between the parameters and survival was assessed using a logistic regression model with stepwise forward selection. A multivariate logistic regression model was carried out using a stepwise selection method to identify the significant factors between the groups. A *p* < 0.05 value was considered statistically significant.

## 5. Conclusions

In conclusion, our data underline that CZA and C/T are important additions to the antimicrobial armamentarium, with good activity against MDR/XDR-PA, and they could be lifesaving in the case of infections due to isolates resistant to all other traditional anti-pseudomonal beta-lactams.

Moreover, considering the different C/T and CZA residual susceptibilities, both agents may be considered complementary therapeutic resources for hard-to-treat PA-related infections in specific clinical and epidemiological settings. 

We believe that these data could contribute to antimicrobial stewardship programs aiding to correctly use these new BLBLICs, considering that real-world experiences designed to compare risk factors for resistance and outcomes in infections with CZA and C/T-resistant MDR-PA strains are scarce.

Greater amounts of larger and prospective studies are needed to understand how C/T and CZA can best be incorporated into clinical practice in order to optimize effectiveness while minimizing the emergence of resistance. Future efforts to solve the dilemma of where to place these new drugs must be made, to support their use in CA-sparing regimens (as Spanish guidelines suggest) or to reserve them for CA-resistant strains treatment only. 

## Figures and Tables

**Table 1:** Comparison of demographic and clinical characteristics between patients colonized/infected with S-C/T + CZA and R-C/T + CZA PA.

Characteristics	Total Population	S-C/T + CZA PA	R-C/T + CZA PA	*p*
Age, M (Q1–Q3), years	67 (59–76)	64 (58.5–75)	74 (66–82)	0.090
Male sex, % (n. of pts)	63.9 (71/111)	60.0 (36/60)	77.7 (21/27)	0.107
**Microbiological Specimen of PA, % (n. of pts)**				
Airways	34.3 (38/111)	38.3 (23/60)	18.5 (5/27)	0.129
Urine	18.9 (21/111)	16.7 (10/60)	33.3 (9/27)	
Blood	7.2 (8/111)	3.3 (2/60)	11.1 (3/27)	
Surgical wound swab/pus	21.6 (24/111)	25 (15/60)	14.8 (4/27)	
Rectal	1.8 (2/111)	1.7 (1/60)	3.7 (1/27)	
Liquor	1.8 (2/111)	3.3 (2/60)	0.0 (0/27)	
Intra-abdominal	7.2 (8/111)	5.0 (3/60)	14.8 (4/27)	
Other	7.2 (8/111)	6.7 (4/60)	3.7 (1/27)	
**Underlying Systemic Disease**				
CCI, mean (±SD)	5.3 (±2.6)	4.7 (±2.5)	6.1 (±2.3)	0.026
CCI 0–3, % (n. of pts)	23.4 (26/111)	31.7 (19/60)	14.81 (4/27)	0.125
CCI 4–6, % (n. of pts)	44.1 (49/111)	40.0 (24/60)	37.0 (10/27)	
CCI ≥ 7, % (n. of pts)	32.4 (36/111)	28.3 (17/60)	48.1 (13/27)	
Prior PA colonization, % (n. of pts)	28.8 (32/111)	27.1 (16/59)	66.6 (18/27)	<0.001
**Hospitalization**				
Time at risk, days, M (Q1–Q3)	22 (7–45)	23.0 (±23.5)	37.3 (±35.4)	0.042
Previous admission in ICU % (n. of pts)	47.7 (52/109)	41.4 (24/58)	55.5 (15/27)	0.222
LOS in ICU, days, M (Q1–Q3)	1 (0–22)	0 (0–17)	4 (0–42)	0.030
**Devices**				
OTI/TT, % (n. of pts)	40.9 (45/110)	37.3 (22/59)	44.4 (12/27)	0.529
CVC, % (n. of pts)	30.9 (34/110)	18.6 (11/59)	40.7 (11/27)	0.029
UC, % (n. of pts)	52.7 (58/110)	40.7 (24/59)	62.9 (17/27)	0.055
CA previous exposure, days, M (Q1–Q3)	0 (0–9)	0 (0–7)	0 (0–12)	0.252
**Prior Antibiotic Usage, % (n. of pts)**				
C/T	7.2 (8/111)	0 (0/60)	11.1 (3/27)	0.009
CZA	0.9 (1/111)	0 (0/60)	3.7 (1/27)	0.134
CAZ	35.1(39/111	28.3 (17/60)	40.7 (11/27)	0.252
P/T	51.3 (57/111)	43.3 (26/60)	55.6 (15/27)	0.291
3-4GC	36.9 (41/111)	28.3 (17/60)	51.8 (14/27)	0.034
FQ	15.3 (17/111)	15 (9/60)	3.7 (1/27)	0.126
**Outcomes**				
LOS, days (±SD)	49 (21–87)	37.5 (19–64)	60 (27–107)	0.061
Crude overall mortality, % (n. of pts)	41.8 (46/110)	38.3 (23/60)	61.5 (16/26)	0.047
Crude attributable mortality %, (n. pts)	46.0 (35/76)	47 (16/34)	60.0 (12/20)	0.263

**Legend:** UTI: urinary tract infection; PJI: prosthetic joint infection; ABSSSI: acute bacterial skin and skin structure infection; IAI: intra-abdominal infection; BSI: blood stream infection; CNSI: central nervous system infection; CCI: Charlson Comorbidity Index; LOS: length of stay; OTI/TT: oro-tracheal intubation/tracheostomy tube; CVC: central venous catheter; UC: urinary catheter; C/T: ceftolozane-tazobactam; CZA: ceftazidime-avibactam; CA: carbapenems; P/T: piperacillin-tazobactam; 3-4GC: third to fourth generation cephalosporins; and FQ: fluoroquinolones.

**Table 2:** Multivariable model of risk factors for the isolation of a R-C/T + CZA PA strain.

Characteristics	R-C/T + CZA PA and S-C/T + CZA PA		
	Adjusted RR (95% CI)		*p*-Value
Age	1.02	(1.02–1.02)	<0.001
CCI 0–3	ref.		
CCI 4–6	1.09	(0.34–3.10)	0.872
CCI ≥ 7	2.30	(1.01–6.21)	0.064
Prior PA colonization	3.69	(1.99–4.65)	<0.001
Time at risk	1.00	(1.01–1.04)	<0.001
UTI	1.56	(1.32–2.90)	<0.001
CA previous exposure	1.73	(1.73–1.74)	<0.001

**Legenda:** CCI: Charlson Comorbodity; UTI: urinary tract infection; and CA: carbapenems.

**Table 3:** Multivariable model of risk factors for 30-day attributable mortality.

Predictors	OR CI (95%)	*p*-Value
**Microbiological specimen of PA**		
PA-related BSI	6.59 (0.65–66.5)	0.110
**Underlying systemic disease**		
CCI 0–3	Ref.	
CCI 4–6	4.85 (0.93–25.07)	0.060
CCI ≥ 7	8.89 (1.52–51.92)	0.015
**Related to hospitalization**		
LOS in ICU, days	1.03 (1.01–1.05)	0.004
**Antibiotic resistance**		
R-C/T + CZA	1.43 (0.48–4.24)	0.511

## Data Availability

The data presented in this study are available on request from the corresponding author.
